# Supplementary material for: Successful captive breeding of a Malayan pangolin population to the third filial generation
Source: Commun Biol. 2021 Oct 21;4:1212. doi: 10.1038/s42003-021-02760-4 (PMC8531396; doi:10.1038/s42003-021-02760-4)
Supplement: Supplementary file 2 — Supplementary Information [file 42003_2021_2760_MOESM2_ESM.pdf]

Successful captive breeding of a Malayan pangolin population to the third filial generation

Dingyu Yan[1#], Xiangyan Zeng[1], Miaomiao Jia[1], Xiaobing Guo[1], Siwei Deng[2], Li Tao[3], Xiaolu Huang[1], Baocai Li[1], Chang Huang[2], Tengcheng Que[4], Kaixiang Li[1], Wenhui Liang[1], Yao Zhao[1], Xingxing Liang[1], Yating Zhong[1], Sara Platto[5], Siew Woh Choo[2,6#]

[1]Guangxi Forestry Research Institute, Nanning 530002, P.R. China

[2]Department of Biology, College of Science and Technology, Wenzhou-Kean University, Wenzhou, Zhejiang, P.R. China

[3]Guangxi Institute of Veterinary Research, Nanning 530001, P.R. China

[4]Guangxi Terrestrial Wildlife Rescue Research and Epidemic Disease Monitoring Centre, Nanning, Guangxi, 530003, P.R., China

[5]Department of Biotechnology, College of Life Sciences, Jianghan University, Wuhan, Hubei, P.R. China

[6]Zhejiang Bioinformatics International Science and Technology Cooperation Centre, Wenzhou-Kean University, Wenzhou, Zhejiang, P.R. China

# = Corresponding authors:

Dingyu Yan, Guangxi Forestry Research Institute, Guangxi, China

Email: Yandy6@126.com

Siew Woh Choo, Wenzhou-Kean University, Zhejiang, China

Email: cwoh@wku.edu.cn

Supplementary Information

Supplementary Table 1. List of 33 wild-caught Malayan pangolins (*Manis javanica*) used in this study. Survival and mortality were recorded until 30 November 2020. WF = Wild female, WM = Wild male. Initial mass is the mass when the pangolin entered our rescue center.

No.	Pangolin ID	Entry date	Initial mass (kg)	Current status	Survival days
1	WF1	11/9/2013	3.3	died on 13/7/2018	1765
2	WF2	10/1/2014	2.3	alive	2514
3	WF3	10/1/2014	2.6	alive	2514
4	WF4	26/1/2014	1.4	died on 26/2/2016	761
5	WF5	4/9/2015	3.7	died on 28/8/2019	1453
6	WF6	4/9/2015	3.5	alive	1912
7	WF7	22/9/2015	3.9	died on 29/1/2016	129
8	WF8	15/12/2015	4.0	alive	1810
9	WF9	15/12/2015	4.3	died on 1/5/2016	136
10	WF10	15/12/2015	3.6	died on 8/2/2018	785
11	WF11	19/1/2016	3.7	died on 5/9/2017	594
12	WF12	19/1/2016	3.5	alive	1775
13	WF13	4/3/2016	3.9	died on 20/11/2016	261
14	WF14	16/4/2016	4.3	died on 4/6/2019	1144
15	WF15	16/4/2016	4.5	died on 12/6/2019	1152
16	WF16	16/4/2016	5.3	alive	1688
17	WM1	13/4/2014	6.7	alive	2421
18	WM2	13/4/2014	7.8	alive	2421
19	WM3	22/9/2014	5.0	alive	2259
20	WM4	22/9/2014	3.1	alive	2259
21	WM5	16/9/2015	3.6	alive	1900
22	WM6	16/9/2015	3.7	alive	1900
23	WM7	22/9/2015	5.6	alive	1894
24	WM8	15/12/2015	2.3	died on 5/3/2019	1174
25	WM9	15/12/2015	4.5	alive	1809
26	WM10	15/12/2015	9.6	alive	1809
27	WM11	19/1/2016	7.2	died on 13/11/2016	298

28	WM12	19/1/2016	8.9	alive	1775
29	WM13	4/3/2016	5.0	died on 29/12/2017	665
32	WM16	16/4/2016	6.5	died on 17/9/2017	519
33	WM17	16/4/2016	9.0	died on 5/10/2017	537

Supplementary Table 2. List of 49 captive-born Malayan pangolins (*Manis javanica*) used in this study and their status. Survival and mortality were recorded until 30 November 2020. ♀ = female, ♂ = male, ? = unknown, FG = First-generation offspring, SG = Second-generation offspring, TG = Third-generation offspring

No.	Pangolin ID	Sex	Date of birth	Current status	Survival days
1	FG1	NA	29/4/2016	died on 30/9/2016	154
2	FG2	♂	1/5/2016	alive	1673
3	FG3	♀	5/6/2016	alive	1638
4	FG4	♀	25/6/2016	died on 8/6/2018	713
5	FG5	♂	4/7/2016	died on 24/5/2019	1054
6	FG6	♀	22/8/2016	died on 16/11/2019	1181
7	FG7	♀	18/10/2016	died on 7/7/2018	627
8	FG8	♂	15/4/2017	died on 18/9/2017	156
9	FG9	♂	19/5/2017	died on 8/2/2020	995
10	FG10	♀	27/5/2017	alive	1282
11	FG11	♂	15/7/2017	alive	1233
12	FG12	♂	12/8/2017	died on 22/8/2017	10
13	FG13	NA	fetal death	fetal death	
14	FG14	NA	20/2/2018	died on 3/3/2018	11
15	FG15.1 (twin)	♀	31/3/2018	died on 6/12/2019	615
16	FG15.2 (twin)	♀	31/3/2018	fetal death	0
17	FG16	♀	19/5/2018	alive	925
18	FG17	♂	25/5/2018	died on 3/6/2018	9
19	FG18	♀	11/6/2018	alive	902
20	FG19	♂	13/7/2018	died on 4/8/2018	22
21	FG20	♂	22/7/2018	died on 28/8/2019	402
22	FG21	♀	25/9/2018	alive	796
23	FG22	♀	12/11/2018	alive	748
24	FG23	♀	5/4/2019	alive	604
25	FG24	♀	22/4/2019	alive	587
26	FG25	♀	19/5/2019	died on 24/5/2019	5
27	FG26	NA	fetal death	fetal death	0
28	FG27	♂	10/11/2019	died on 13/11/2019	3
29	FG28	♀	20/3/2020	died on 4/6/2020	76
30	FG29	♀	17/5/2020	alive	197
31	FG30	♀	23/9/2020	died on 29/9/2020	6
32	SG1	♀	15/2/2018	died on 19/3/2018	32
33	SG2	♀	10/3/2018	died on 31/7/2018	143

34	SG3	NA	4/4/2018	died during birth	0
35	SG4	♀	6/6/2018	alive	907
36	SG5	♂	26/7/2018	died on 30/7/2018	4
37	SG6	♀	6/10/2018	alive	785
38	SG7	♂	12/2/2019	alive	656
39	SG8	♂	1/6/2019	alive	547
40	SG9	NA	13/6/2019	died on 29/12/2019	199
41	SG10	NA	26/8/2019	died on 29/8/2019	3
42	SG11	♀	8/3/2020	alive	267
43	SG12	♀	8/4/2020	alive	236
44	SG13	♀	19/5/2020	died on 6/6/2020	18
45	SG14	♂	1/7/2020	alive	152
46	SG15	♂	29/10/2020	alive	32
47	TG1	♀	12/7/2019	alive	506
48	TG2	♀	1/7/2020	died on 24/7/2020	23
49	TG3	♂	20/7/2020	died on 24/7/2020	4

Supplementary Table 3: Basic information of male Malayan pangolins (*Manis javanica*) used for mating**.**

**Pangolin ID**	**Time of entry/birth**	**Mass during entry (kg)**	**Mass during Mmating (kg)**
WM6	16/9/2015	3.7	7.0–8.3
WM8	15/12/2015	2.3	7.0–8.5
WM9	15/12/2015	4.5	5.0–6.0
SG7	12/2/2019	-	4.5

Supplementary Table 4. Sexual maturity of captive-born Malayan pangolins (*Manis javanica*). Four captive-born female offspring successfully mated with male pangolins and conceived even before they separated from their mothers.

ID	Date of birth (offspring used for mating)	Date of conception	Date of separation from mother	Date of giving birth to next-generation offspring
FG10	27/5/2017	20–23/1/2018	10/6/2018	26/7/2018
FG15.1	31/3/2018	8–10/12/2018	3/4/2019	13/6/2019
SG4	6/6/2018	8–11/1/2019	4/4/2019	12/7/2019
FG16	19/5/2018	1/3/2019	28/3/2019	26/8/2019

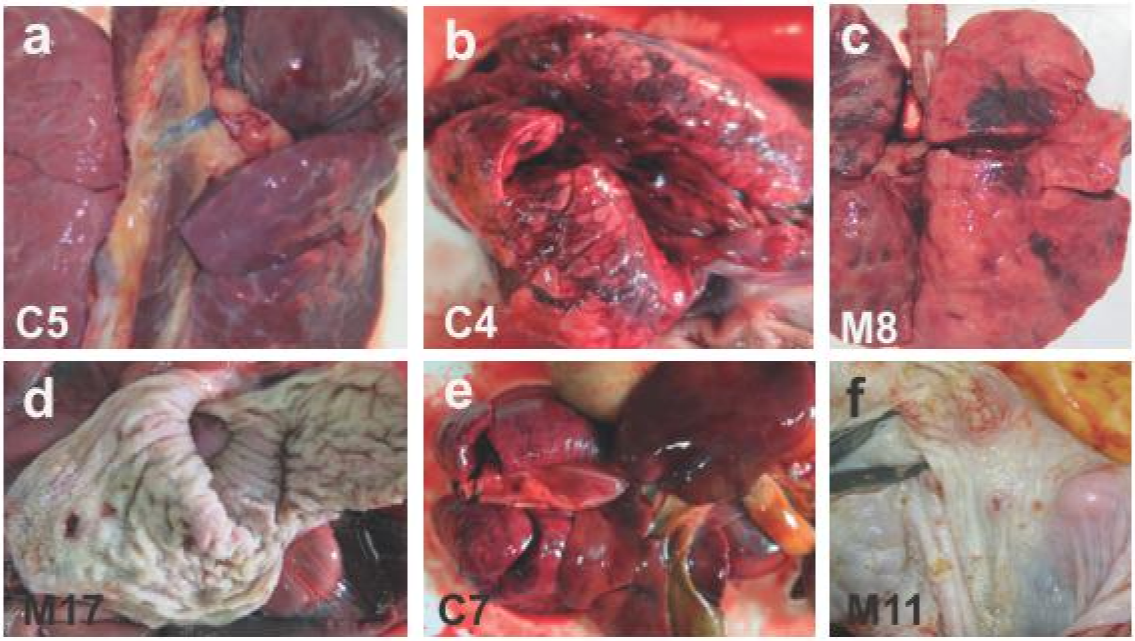

Supplementary Figure 1. Necropsy of dead pangolins. (a) FG5 showed pulmonary haemorrhage and hepatisation. (b) FG4 had multiple hemorrhagic plaques in the lungs. (c) WM8 had pulmonary haemorrhage, hepatisation, and left lung atrophy. (d) There were gastric ulcer foci detected in WM17. (e) Pulmonary haemorrhage and hepatisation were observed in FG7. (f) There were gastric ulcer foci observed in WM11. (Photos: Dingyu Yan).
