# Supplementary material for: Successful captive breeding of a Malayan pangolin population to the third filial generation
Source: Commun Biol. 2021 Oct 21;4:1212. doi: 10.1038/s42003-021-02760-4 (PMC8531396; doi:10.1038/s42003-021-02760-4)
Supplement: Supplementary file 3 — Description of Additional Supplementary Files [file 42003_2021_2760_MOESM3_ESM.pdf]

Description of Additional Supplementary Files

File name: Supplementary Video 1
Description: The tail of a mother pangolin serves as a cradle for her cub.

File name: Supplementary Video 2
Description: Mating of captive-born pangolin FG6. On 16 August 2017, WM6 mated with FG6 for 50 seconds. FG6 was 359 days old and weighed 3.6 kg. The first litter of cub SG1 was born on 15 February 2018. The gestation period of this litter was 185 or 184 days, as mating was completed on 15–16 August 2017.

File name: Supplementary Video 3
Description: Mating of captive-born pangolin FG10. Offspring FG10 and WM6 mated on 21 January 2018. At this time, FG10 was born at 239 days old and weighed 3.58 kg. The same year on 26 July, she gave birth to cub SG5, which had a gestation period of 187 or 184 days because she mated on 20–21 and 23 January 2018.

File name: Supplementary Data 1
Description: Records of cage mating and reproduction of wild female Malayan pangolins (Manis javanica). WF = Wild female, WM = Wild male, FG = First-generation offspring, ♂= male, ♀= female. Maternity data were recorded until 30 November 2020.

File name: Supplementary Data 2
Description: Mating and reproduction of captive-born Malayan pangolin (Manis javanica) offspring. WF = Wild female, WM = Wild male, FG = First-generation offspring, SG = Second-generation offspring, TG = Third-generation offspring, ♂= male, ♀ = female. Maternity records were recorded until 30 November 2020.
